# Association of *BMP15* and *GDF9* Gene Polymorphisms with Litter Size in Hu Sheep

**DOI:** 10.3390/genes16020168

**Published:** 2025-01-28

**Authors:** Yuting Zhang, Haitao Wang, Tingting Li, Na Zhang, Jieran Chen, Hengqian Yang, Shiyu Peng, Runlin Ma, Daxiang Wang, Qiuyue Liu, Yuanyuan Wang

**Affiliations:** 1 School of Life Science, Bengbu Medical University, Bengbu 233000, China; zhangyut09@163.com (Y.Z.); pengsy@163.com (S.P.); 2 State Key Laboratory of Molecular Developmental Biology, Institute of Genetics and Developmental Biology, Chinese Academy of Sciences, Beijing 100101, China; wanght@genetics.ac.cn (H.W.); tingli0704@163.com (T.L.); zhangna4520@163.com (N.Z.); 17852401863@163.com (J.C.); yhq17806253397@163.com (H.Y.); rlma@genetics.ac.cn (R.M.); 3 Jiangsu Qianbao Animal Husbandry Co., Ltd., Yancheng 224050, China; wdx911012@163.com; 4 Anhui Provincial Key Laboratory of Tumor Evolution and Intelligent Diagnosis and Treatment, Bengbu Medical University, Bengbu 233000, China

**Keywords:** *BMP15*, *GDF9*, polymorphisms, litter size, Hu sheep

## Abstract

(1) Background: Litter size is one of the most important economic traits of sheep. The *FecB* locus has been extensively studied due to its significant impact on litter size in Hu sheep, and *BMP15* and *GDF9* have also been reported as major genes associated with litter size in sheep. This study aimed to identify variants of *BMP15* and *GDF9* and perform an association analysis of these variants with litter size in the Hu sheep breed. (2) Methods: In this study, exons of the *BMP15* and *GDF9* genes were fully sequenced to identify polymorphisms in Hu sheep. Population genetic parameters and haplotype frequencies were estimated, and an association analysis between these polymorphic loci and litter size was performed. Additionally, the protein structures of the wild-type and mutated *BMP15* and *GDF9* genes were predicted. (3) Results: The polymorphisms of the *BMP15* and *GDF9* genes were investigated within their exon regions, revealing mutations at four previously reported sites: *BMP15* c.31_33CTTdel and *GDF9* (G2, G3, and G4) in Hu sheep, with no novel variants were detected. Genetic analysis indicated that the *GDF9*-G3 and *GDF9*-G4 loci have low polymorphisms, whereas the *BMP15* c.31_33CTTdel and the *GDF9*-G2 locus are moderately polymorphic. The mutation sites in the *BMP15* and *GDF9* genes were under Hardy–Weinberg equilibrium. Association analysis revealed that the *BMP15* c.31_33CTTdel and *GDF9* (G2, G3, and G4) mutations are not associated with litter size in Hu sheep. Protein structure prediction indicated that the mutations in *BMP15* and *GDF9* resulted in alterations to their tertiary structures. (4) Conclusions: In this study, four reported mutations in the *BMP15* and *GDF9* genes can also be detected in the Hu sheep breed. In these mutations, the G2 and G3 mutations of *GDF9* did not alter the amino acid sequence, while the *BMP15* c.31_33CTTdel mutation and the *GDF9* G4 mutation resulted in protein structure alteration. Furthermore, the BMP15 c.31_33CTTdel mutation and the *GDF9* mutations (G2, G3, G4) were associated with an increased tendency in litter size. However, no significant difference was observed (*p* > 0.05). This study provides valuable insights for improving the lambing performance of Hu sheep.

## 1. Introduction

Sheep (Ovis aries) are important agricultural economic animals [1]. The productive efficiency of the sheep industry largely depends on reproductive performance [2,3]. Most Chinese local sheep breeds, including the Tibetan and Mongolian sheep breeds, typically produce single lambs. Only a few sheep breeds, such as the Hu sheep and the Small-tailed Han sheep, can deliver multiple lambs per litter [4]. Hu sheep, a distinctive local breed in China, demonstrate outstanding reproductive traits, including early sexual maturation, high fecundity, and year-round continuous estrous cycles.

In the past decade, the Chinese government has significantly increased the proportion of domestic sheep raised in housed or semi-housed systems to 70% of total production. This intensive approach incorporates industrial practices into traditional farming, alleviating constraints on environmental resources. Within this large-scale farming framework, over 90% of enterprises have chosen the Hu sheep as their primary maternal breed.

The Hu sheep, originally raised in the Taihu Basin, is a descendant of Mongolian sheep. It was introduced to southern China during the Song Dynasty by northern settlers and subsequently adapted to its new environment. The Hu sheep is predominantly white, exhibiting little to no pigmentation. Both rams and ewes are polled. Its physiological traits make it particularly well-suited for intensive housed feeding systems [5]. First, the Hu sheep boasts exceptionally high fecundity, arguably one of the highest among sheep breeds worldwide. The lambing percentage exceeds 200%, and the annual birth rate averages 300%. Additionally, female Hu sheep reach sexual maturity at 4 to 5 months of age, while males mature at 5 to 6 months, allowing for year-round breeding and continuous productivity [6,7]. Currently, the majority of Hu sheep enterprises operate on a large scale, with herds exceeding 10,000 ewes. Breeding efforts within these enterprises primarily focus on enhancing meat production, as the increased economic value of meat further underscores the importance of reproductive traits. The average weight of an adult male Hu sheep ranges from 70 to 88 kg, while adult females typically weigh between 45 and 56 kg. The slaughter age for Hu sheep is generally between 6 and 7 months [8].

Litter size is influenced by multiple factors, including genetics, environmental conditions, and nutritional status, in which genetics serves as the most critical determinant [9]. At present, studies have identified several major genes that influence litter size in sheep by utilizing different sheep populations and breeds. The known major genes whose mutations can increase ovulation rate are *BMPR1B*, *BMP15*, and *GDF9*, all of which belong to the transforming growth factor beta (TGFβ) superfamily [10]. Previous studies identified a mutation locus (A746G) in the Booroola Merino breed, designated as *FecB* by the International Committee on Sheep and Goat Genetics. This mutation is located in the coding sequence region (CDS) of the *BMPR1B* on chromosome 6, resulting in an amino acid substitution from glutamine (Glu) to arginine (Arg) at position 249 (Q249R) [11,12]. The *FecB* in sheep has been demonstrated to play a crucial role in increasing ovulation numbers [13]. The Hu sheep is a native Chinese breed well known for its high fecundity. The frequency of the G allele in the *FecB* mutation of Hu sheep can reach 95%, which may explain the breed’s elevated reproductive rate. Currently, the *FecB* locus is widely used as a molecular marker of litter size in Hu sheep [4,14,15,16]. *BMP15*, located on the X chromosome in sheep, plays a critical role in ovarian folliculogenesis [17]. Numerous *BMP15* mutations have been identified in sheep, including B1, B2 (FecX^G^), B3, B4 (FecX^B^) [18], FecX^I^, FecX^H^ [19], FecX^L^ [20], FecX^R^ [21], FecX^Gr^, and FecX^O^ [22]. Previous studies in sheep indicated that mutants of *BMP15* can increase ovulation rate in heterozygotes, while homozygous mutants display primary ovarian failure and lead to infertility, such as FecX^I^, FecX^H^, FecX^G^, etc. [10,20,21,22,23,24]. It is noteworthy that the homozygous ewes of FecX^R^ and FecX^O^ mutations also had an increased ovulation rate without becoming sterile. *GDF9* is located on chromosome 5 in sheep and plays a crucial role in the growth and differentiation of follicles. More than a dozen *GDF9* mutations have been identified in sheep, including G1, G2, G3, G4, G5, G6, G7, G8, FecG^G^, FecG^E^, FecG^A^, etc. [25,26,27]. In most of these mutations, heterozygous ewes carrying mutations in the *GDF9* gene exhibit an increased ovulation rate and litter size. In contrast, homozygous ewes become infertile due to ovarian hypoplasia and failure of primary folliculogenesis.

Owing to the low heritability of litter size in sheep, traditional breeding methods have limited efficiency in increasing litter size. However, marker-assisted selection (MAS) has the potential to increase lambing rates within a shorter timeframe. Following the discovery of the *FecB*, *BMP15,* and *GDF9* genes have emerged as crucial determinants of litter size in sheep [28], being used to explain the increased litter size and/or ovulation rate in a variety of breeds/strains, including Inverdale, Cambridge, Belclare, and Lacaune sheep. To date, numerous studies have been conducted on the *BMP15* and *GDF9* genes across various sheep breeds. However, only a few mutations in these genes associated with litter size have been identified in domestic sheep breeds [29]. In this study, we identify the polymorphisms of the *GDF9* and *BMP15* genes in Hu sheep and investigate the association between these polymorphic loci and litter size in the Hu sheep population. Four previously reported mutations, including *BMP15* c.31_33CTTdel and *GDF9* (G2, G3, and G4), have been assessed for their genetic diversity within the breed, and the association analysis of those mutations with litter size was performed in Hu sheep. This study provides valuable insights for improving the lambing performance of Hu sheep.

## 2. Materials and Methods

### 2.1. Ethics Statement

All experimental procedures adhered to the ethical standards set forth by the Animal Advisory Committee at the Institute of Genetics and Developmental Biology, Chinese Academy of Sciences (Approval No. AP2022015-C1).

### 2.2. Experimental Animals

Hu sheep were produced and raised by Jiangsu Qianbao Animal Husbandry Co., Ltd. (Yancheng, China). Over a period of five years (2020–2024), data were collected from a nucleus flock with 5188 Hu sheep ewes. All breeding data were recorded using the Pedigree and Sheep Management System with electronic tags. This system allows for the viewing or printing of up to a three-generation pedigree, and its powerful search function enables the retrieval of sheep data at any time. Mating, pregnancy, lactation, litter size, weight, and other phenotypes can be kept for each sheep. Full offspring statistics can be viewed for all sheep used for breeding. The number of lambs per litter was documented, including counts for the first, second, and third litters, with a range of 1 to 6 lambs per litter. A total of 322 healthy adult ewes, aged 4–5 years, were sourced from this Hu sheep nucleus flock for use in this study. The ewes were housed in the same sheds, and supplements (silage and peanut seedlings) were provided as required by commercial standards. All ewes underwent artificial insemination for breeding. They were vaccinated against clostridial diseases and received preventative treatments. Lambs were weighed at birth and tagged, typically within 12 h of birth, with continuous measurements taken for six months post-lambing.

Approximately 5 mL of blood was collected from the jugular vein of each ewe using EDTA blood collection tubes and stored at −80 °C for subsequent DNA extraction.

### 2.3. DNA Extraction

The genomic DNA of these ewes was extracted with a blood genomic DNA extraction kit (Tiangen Biotech Co., Ltd., Beijing, China). The concentration and quality of genomic DNA were assessed using 1% agarose gel electrophoresis. The concentration and purity of the DNA were quantified with an ultramicro spectrophotometer (NanoDrop One, Thermo Scientific, Waltham, MA, USA). The samples were then stored at −20 °C until further use.

### 2.4. Primer Design and PCR Amplification

The primer design for PCR amplification of *BMP15* and *GDF9* was conducted using NCBI BLAST (https://blast.ncbi.nlm.nih.gov/Blast.cgi, accessed on 1 September 2023), and the reference genome version is ARS-UI Ramb v3.0. The target fragment encompassed the exonic regions of both genes. All primers were synthesized by Sangon Biotech (Shanghai) Co., Ltd. (Shanghai, China). Table 1 provides comprehensive details of the *BMP15* and *GDF9* primers.

### 2.5. Genotyping and DNA Sequencing

The polymerase chain reaction (PCR) was performed in a 25 µL reaction mixture, which included 1 µL of genomic DNA, 1 µL each of forward and reverse primers, 12.5 µL of 2× Taq Master Mix, and 9.5 µL of ddH2O. The thermal cycling protocol consisted of an initial denaturation step at 95 °C for 3 min, followed by 35 cycles consisting of denaturation at 95 °C for 30 s, annealing at 59 °C for 30 s, and extension at 72 °C for 1 min, concluding with a final extension at 72 °C for 10 min. Subsequently, the PCR products were sequenced by Sangon Biotech to confirm the presence of the mutation.

### 2.6. Prediction of Protein Structure

The protein tertiary structure prediction of wild-type and mutant BMP15 and GDF9 was performed using a homology modeling approach through the Swiss-Model platform.

### 2.7. Statistical Analysis

Hardy–Weinberg equilibrium of *BMP15* and *GDF9* in the Hu sheep population was assessed using the chi-square test. We calculated population genetic indices, including expected heterozygosity (He), the number of effective alleles (NE), and polymorphic information content (PIC), as well as genotype and allele frequencies, following the methodology outlined by Nei [30]. The litter size of different parities was tested for normal distribution using the Kolmogorov–Smirnov test and did not satisfy normal distribution (*p* < 0.05) (Supplementary Material S1 and S2). We employed the Kruskal–Wallis test to investigate the relationship between the *BMP15* c.31_33 polymorphisms and the number of lambs per litter in a sample of 322 Hu sheep. Data are shown as mean and median. The results were considered significant when *p* < 0.05. Additionally, we examined the associations between mutations in the *GDF9* gene (G2, G3, and G4) and the number of lambs per litter.

## 3. Results

### 3.1. Amplification of the BMP15 and GDF9

**Figure 1:**
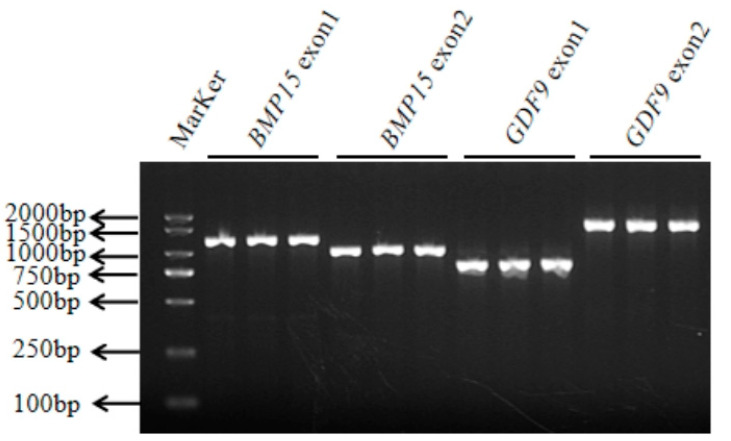
The specific fragment of the *BMP15* and *GDF9* genes. The electrophoresis results of the PCR-amplified fragments of the *BMP15* and *GDF9* genes.

Four pairs of primers of the *BMP15* gene (Exon 1 and Exon 2) and the *GDF9* gene (Exon 1 and Exon 2) were designed according to reference sequences. The amplification sizes for *BMP15*-Exon 1, *BMP15*-Exon 2, *GDF9*-Exon 1, and *GDF9*-Exon 2 were 1258 bp, 1080 bp, 813 bp, and 1656 bp, respectively. The amplified fragment (Figure 1) was consistent with the target fragment.

### 3.2. Identification of Mutations in the BMP15 and GDF9 Genes

**Figure 2:**
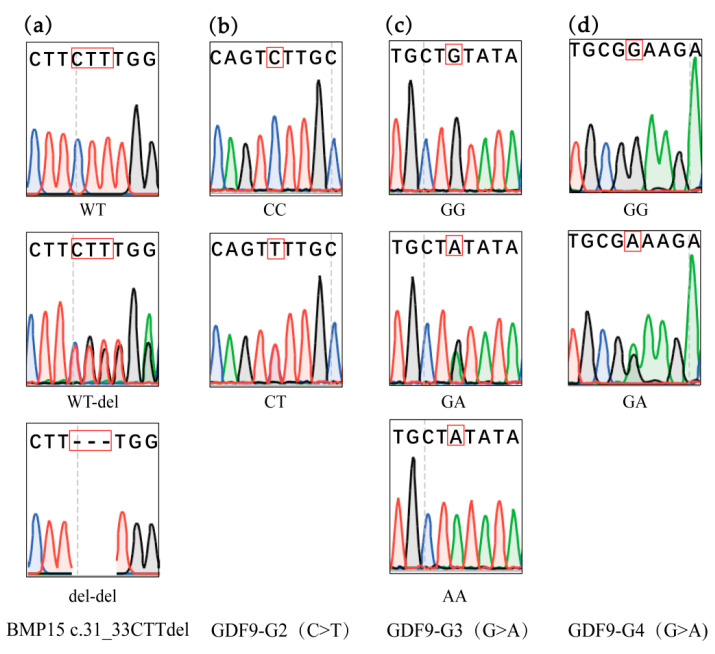
Mutations of *BMP15* and *GDF9* by genotyping; (**a**) genotype of the *BMP15* c.31_33CTTdel mutation in Hu sheep; (**b**) genotypes of the *GDF9*-G2 site in Hu sheep; (**c**) genotypes of the *GDF9*-G3 site in Hu sheep; (**d**) genotypes of the *GDF9*-G4 site in Hu sheep. The genotypes of identified mutations were marked by red boxes.

The polymorphisms of the *BMP15* and *GDF9* genes were investigated within their exon regions, revealing mutations at four previously reported sites: *BMP15* c.31_33CTTdel and *GDF9* (G2, G3, and G4) in Hu sheep, with no novel variants detected. Specifically, a deletion mutation of CTT was identified at positions 31–33 in the coding region of the *BMP15* gene, referred to as *BMP15* c.31_33CTTdel. In Hu sheep, this mutation resulted in three genotypes: WT, WT/del, and del/del. For the *GDF9* gene, the reported mutations included G2, G3, and G4 (see Figure 2). Table 2 provides a comprehensive overview of the polymorphism information for the four mutations in the *BMP15* and *GDF9* genes in Hu sheep.

### 3.3. Protein Structure Prediction

**Figure 3:**
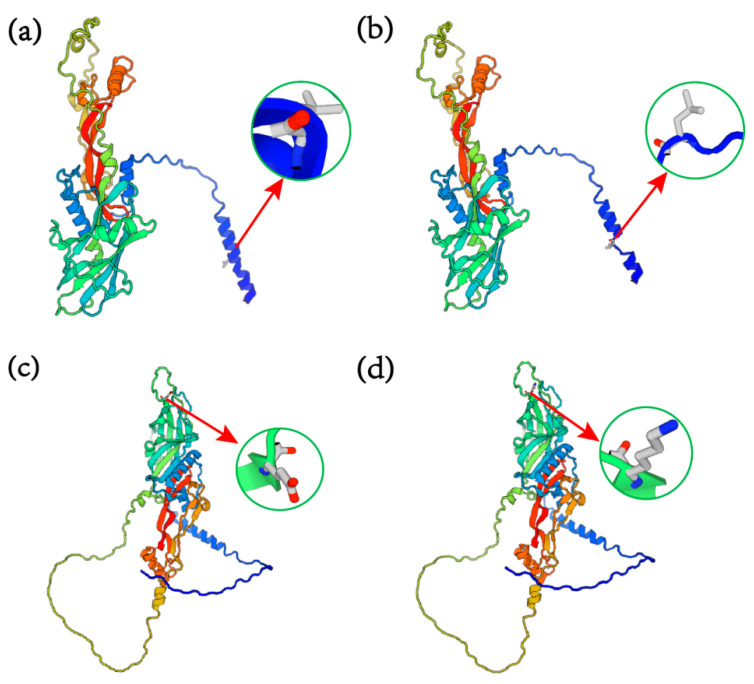
The tertiary structures of wild-type and mutant BMP15 and GDF9 proteins. (**a**) The predicted tertiary structures of the wild-type BMP15 protein; (**b**) the predicted tertiary structures of *BMP15* after the *BMP15* c.31_33CTTdel mutation; (**c**) the predicted tertiary structures of wild-type *GDF9*; (**d**) the predicted tertiary structures of GDF9 after the mutant *GDF9*-G4 site.

We conducted predictions of the tertiary protein structures of wild-type and mutant forms of both *BMP15* c.31_33CTTdel and *GDF9* G4 (G > A), respectively. As shown in Figure 3, despite no significant alterations in the overall spatial configuration of the proteins, the BMP15 protein experienced a loss of leucine at the 11th amino acid position (Figure 3a,b). The mutant GDF9 protein showed the substitution of lysine with glutamate at the 241st amino acid position (Figure 3c,d). These modifications resulted in changes in the tertiary structures of both BMP15 and GDF9.

### 3.4. Genetic Analysis of Hu Sheep Population

A population genetic analysis was conducted on four mutation sites within the *BMP15* and *GDF9* genes (Table 3). The results indicated that the *GDF9*-G2 and *GDF9*-G4 loci exhibited low polymorphisms (PIC < 0.25), while the *BMP15* c.31_33CTTdel and the *GDF9*-G3 locus demonstrated moderate polymorphisms (0.25 < PIC < 0.5). Furthermore, the chi-square test revealed that the *BMP15* c.31_33CTTdel locus and the G2, G3, and G4 loci of *GDF9* were under Hardy–Weinberg equilibrium in Hu sheep (*p* > 0.05).

### 3.5. Association Analysis of BMP15 and GDF9 Mutations with Litter Size Across Different Parities in Hu Sheep

An evaluation of the association between *BMP15* c.31_33CTTdel locus with litter size across different parities was performed in the nucleus flock of Hu sheep (Table 4). The analysis revealed no statistically significant differences in litter size among the various genotypes of the *BMP15* c.31_33CTTdel mutation (*p* > 0.05). In terms of litter size across different parities, ewes with the del/del genotype displayed a numerically higher litter size compared to those with the wild-type (WT) and WT-del genotypes, except in the third parity; however, these differences were not statistically significant (*p* > 0.05).

An evaluation of the association between G2, G3, and G4 loci of *GDF9* with litter size across different parities was also performed in the nucleus flock of 195 Hu sheep (Table 5). Analysis of variance revealed no significant association between the polymorphisms at the G2, G3, and G4 loci with litter size in all parities (*p* > 0.05). Specifically, although the litter sizes for the *GDF9*-G2, G3, and G4 mutant or heterozygous genotypes were higher than those of the wild type, these differences did not achieve statistical significance.

## 4. Discussion

Litter size is a crucial economic indicator in sheep breeding and has been extensively studied in worldwide sheep breeds. Currently, *BMPR1B*, *BMP15*, and *GDF9*, which are all members of the transforming growth factor β (TGFβ) superfamily, are the reported major genes associated with litter size in sheep. Previous studies have reported that the *BMPR1B* gene is located in a region under strong selective sweeps in the Hu sheep breed [5,8,15,32]. It also has been shown that Hu sheep carry two major mutations affecting litter size, including *FecB* [4]. From our findings in the Hu sheep nucleus flock, the allele frequency of the *FecB* mutant was over 0.85. However, it has been found that the ewes carrying homozygous *FecB* locus still produced single offspring, and variations in lambing numbers persist in different parities [15]. The molecular mechanisms underlying exceptional reproductive performance in Hu sheep remain extensively studied. *BMP15* and *GDF9* have been repeatedly identified as major genes influencing the ovulation rate in foreign sheep breeds. The aim of this study was to identify the novel variants of *BMP15* and *GDF9* in Hu sheep breeds and investigate the association between these polymorphic loci and litter size in the Hu sheep population. This may offer new perspectives for molecular breeding in the Hu sheep breed.

In sheep, *BMP15* and *GDF9* are exclusively expressed in oocytes and serve as essential growth factors for follicular development. These factors exert a synergistic effect on the number of ovulations in sheep [33]. It has been proposed that the primary functions of *BMP15* and *GDF9* are to regulate the sensitivity of follicle-stimulating hormone (FSH) in secondary and antral follicles, help prevent premature cell differentiation, and ensure the production of a normal number of preovulatory follicles [34]. During the origination of follicular development, *BMP15* regulates granulosa cell proliferation. In the later stages of follicular development, *BMP15* is believed to influence the differentiation of granulosa cells by inhibiting the expression of FSH, thereby controlling follicular maturation. Furthermore, *BMP15* is involved in regulating cumulus expansion and oocyte composition during the preovulatory period [34,35]. *GDF9* is expressed during the type I follicular phase and plays a crucial role in regulating early follicle development through paracrine signaling. It not only promotes the proliferation of granulosa cells but also induces cumulus expansion and cytokine synthesis. Furthermore, *GDF9* regulates hormone secretion in ovine cumulus cells, thereby maintaining the stability of the follicular microenvironment [36,37]. *BMP15* and *GDF9* can form a heterodimer that collaboratively activates the Smad signaling pathway by interacting with bone morphogenetic protein receptor type II (BMPR-II) and protein receptor type I (BMPR1B). This pathway is essential for regulating oocyte maturation and ovulation, which also influences litter size [38,39,40].

*BMP15* serves as a crucial signaling molecule for the development of ovarian follicles and plays a significant regulatory role in the growth and differentiation of early oocytes. Initially identified in Romney sheep, this gene was designated as the Inverdale gene (FecX^I^) [18]. Subsequent research has uncovered other *BMP15* mutations, including FecX^G^, FecX^H^, FecX^B^, FecX^L^, FecX^R^, and FecX^Bar^. However, these mutations are rarely observed in Chinese local sheep breeds [41]. In this study, a deletion of the CTT sequence at positions 31–33 of exon 1 was identified in the *BMP15* gene of Hu sheep, resulting in the loss of a leucine residue in the *BMP15* protein. Numerous studies have reported the c.31_33CTTdel mutation in the *BMP15* gene across various sheep breeds worldwide. These studies describe leucine-deletion polymorphisms of two leucine codons (CTT) at amino acid positions 10 and 11 in sheep, while another study only reported one amino acid change [18,24,31]. Specifically, the CTT deletion mutation in exon 1 of the *BMP15* gene was observed in Chinese Small-tailed Han sheep by analyzing a total of 240 ewes each with a twin litter size, where a significant association was found between litter size and the *BMP15* gene deletion (*p* < 0.05). On average, homozygotes and heterozygotes produced 0.5 and 0.3 more lambs per litter compared to the wild type, respectively [42]. Meanwhile, one study conducted an association analysis examining the relationship between litter size and the *BMP15* c.31_33CTTdel mutation in a sample of 325 purebred Ujimqin ewes and found the c.31_33CTTdel mutation in the *BMP15* gene was significantly associated with litter size in Ujimqin sheep [43]. The Small-tailed Han sheep, Ujimqin sheep, and Hu sheep are all classified as Mongolian sheep breeds. The c.31_33CTT deletion has been found to be significantly associated with litter size in both the Small-tailed Han and Ujimqin sheep. This may be attributed to the deletion of leucine in the N-terminal signal peptide region, which is hypothesized to influence *BMP15* secretion and affect litter size. In this study, three genotypes of the c.31_33CTT deletion were identified, and the association analysis revealed no significant differences in litter size among these genotypes (*p* > 0.05). The impact of the *BMP15* c.31_33CTTdel mutation on litter size varies across different breeds [31]. Furthermore, within this study, the sample size for the del-del genotype is notably limited, and the distribution of the three genotypes is imbalanced, which may influence the association result.

The *GDF9* gene, a member of the transforming growth factor superfamily, has been extensively studied in sheep. Research has shown that the timing and pattern of *GDF9* gene expression in ruminant oocytes correspond with the initiation and maintenance of folliculogenesis, indicating its critical role in regulating early follicle and oocyte development [44]. In sheep, there are more than a dozen identified mutations of *GDF9*, including G1, G2, G3, G4, G5, G6, G7, G8, and FecG^H^. In a domestic study, researchers identified a mutation site g.46547645T > G, in the promoter region of the *GDF9* gene in Mongolian sheep, which exhibited a significant correlation with the litter size [45]. They performed whole-coding region sequencing of the *GDF9* gene in Shandong Central mutton sheep and discovered two SNPs g.41768501(A > G) and g.41768485(G > A) that were associated with litter size [46]. The *GDF9* mutations G1 to G8 were identified in the prolific sheep breeds Belclare and Cambridge, with G1 located on *GDF9* exon 1 and the others on exon 2. Among these mutations, G2, G3, and G5 do not result in changes in amino acids. The remaining five mutations—G1, G4, G6, G7, and G8—cause amino acid changes. Specifically, G1, G4, G6, and G7 are all G > A mutations that occur at the furin processing site or before the unprocessed protein, making it unlikely to affect the mature active coding region. However, G8 changes serine to phenylalanine at residue 395 and replaces the uncharged polar group with a nonpolar group at residue 77 in the mature coding region, which may alter the function of ovine *GDF9* [17,47]. In Egyptian sheep, the number of litter sizes by *GDF9*-G4 mutant heterozygous ewes was significantly higher than that of wild-type homozygous ewes (*p* ≤ 0.05). The polymorphisms of *GDF9* may influence the lambing rate in sheep. The G1 and G8 mutations have been identified in Kazakhstan meat-wool sheep, with a distinct differentiation observed between the wild type and G1 mutation carriers, potentially correlating with variations in litter size [48]. The variants *GDF9* g.41768501 A > G and g.41768485 G > A were found to be significantly associated with litter size in Luzhong sheep [49]. Several researchers have identified three mutations in the *GDF9* gene of Iranian Afshari sheep, designated as G2, G3, and G4. Although the G4 mutation is unlikely to affect the protein activity of *GDF9*, it may be linked to increased fecundity in Iranian sheep [47]. Several studies have identified the *GDF9* G2 mutation in Small-tailed Han sheep, which has no influence on litter size, a finding that aligns with our research outcomes. In this study, G2, G3, and G4 mutations within the *GDF9* gene can also be found in the Hu sheep population. It was shown that litter size increased following the mutation, but none of these mutations showed a significant association with the litter size in our nucleus flock of Hu sheep. The analysis of the tertiary structure of the GDF9 protein showed that neither the G2 nor the G3 mutations caused any structural changes. In contrast, the G4 mutation led to a substitution of glutamic acid (Glu) with lysine (Lys) at the 241st amino acid position. However, this alteration did not affect the activity of the mature GDF9 protein.

## 5. Conclusions

In this study, four known mutation sites were identified in the *BMP15* and *GDF9* genes in our nucleus flock of Hu sheep population. Among these, the *GDF9* G2 and G3 mutations did not affect the amino acid sequence. The *BMP15* c.31_33CTTdel mutation led to the deletion of leucine at the eleventh amino acid, thereby altering the protein structure of BMP15. The *GDF9*-G4 mutation at position 241 replaced glutamic acid with lysine, resulting in a structural change to the protein. Furthermore, the *BMP15* c.31_33CTTdel mutation and the *GDF9* mutations (G2, G3, and G4) were associated with an increased tendency of litter size, but there was no significant difference.

## Figures and Tables

**Table 1:** Sequences and characteristics of primers used for amplifying the *BMP15* and *GDF9* genes in Hu sheep.

Primer Name	Primer Sequence(5′-3′)	Location	Amplified Fragment/bp
*BMP15*-1F	TAGGGTGGGAACAGGAGGC	*BMP15*-Exon1	1258
*BMP15*-1R	TCAGGACAGCTAAGGAGAGTC		
*BMP15*-2F	TTTACCGCCATCAGCTTCACC	*BMP15*-Exon2	1030
*BMP15*-2R	ACCCCAAACCGTCTAGATCC		
*GDF9*-1F	CAGCTAAGCATCCTTAAGGTCT	*GDF9*-Exon1	813
*GDF9*-1R	TGACCCTGGACAAGATGCT		
*GDF9*-2F	TCCCCACCAAAGCTATTCTGA	*GDF9*-Exon2	1656
*GDF9*-2R	CCTCTCCCTCTCAAATAACCAT		

**Table 2:** Mutations of *GDF9* and *BMP15* in Hu sheep.

Gene	Variant	Base Change	Coding Base (bp)	Coding Residue (aa)	Amino Acid Change
*BMP15*	c.31_33CTTdel	CTTdel	31–33	11	Leu(L)deletion
*GDF9*	G2	C > T	471	157	UnchangedVal(V)
	G3	G > A	477	159	UnchangedLeu(L)
	G4	G > A	721	241	Glu(E) > Lys(K)

**Table 3:** Genotype, allele frequency, and genetic diversity of the *BMP15* Gene (c.31_33 CTTdel) and *GDF9* gene (G2, G3, and G4 loci) in Hu sheep.

Locus	Genotype Frequency			Allele Frequency		Diversity Parameter			
						PIC	HE	NE	*p*-Value
*BMP15* c.31_33 CTTdel [31]	WT	WT_del	del_del	WT	del	0.28	0.33	1.49	0.09
	0.64	0.3	0.06	0.79	0.21				
*GDF9*-G2(C > T) [18]	CC	CT	TT	C	T	0.02	0.02	1.02	0.88
	0.98	0.02	0.00	0.99	0.01				
*GDF9*-G3(G > A) [18]	GG	GA	AA	G	A	0.35	0.45	1.81	0.06
	0.41	0.51	0.08	0.66	0.34				
*GDF9*-G4(G > A) [18]	GG	GA	AA	G	A	0.13	0.14	1.17	0.24
	0.84	0.16	0.00	0.92	0.08				

Note: HWE: Hardy–Weinberg equilibrium; He: expected heterozygosity; NE: effective allele numbers; PIC: polymorphism information content. The classification was conducted according to the PIC values (PIC < 0.25, low polymorphism; 0.25 < PIC < 0.5, moderate polymorphism; and PIC > 0.5, high polymorphism).

**Table 4:** The association analysis between *BMP15* gene polymorphisms and litter size in Hu sheep.

Locus	Parity	Genotype	Number of Samples	Mean (Median)	H-Value	*p*-Value
*BMP15* c.31_33CTTdel [31]	1st	WT	207	2.493 (2.000)	0.070	0.997
		WT-del	96	2.479 (2.000)		
		del-del	19	2.579 (2.000)		
	2nd	WT	171	2.350 (2.000)	1.121	0.571
		WT-del	82	2.293 (2.000)		
		del-del	16	2.500 (2.500)		
	3rd	WT	137	2.453 (2.000)	4.933	0.085
		WT-del	71	2.241 (2.000)		
		del-del	15	2.267 (2.000)		
	Average	WT	207	2.423 (2.330)	1.162	0.559
		WT-del	96	2.330 (2.330)		
		del-del	19	2.491 (2.330)		

Note: 1st parity: the first parity; 2nd parity: the second parity; 3rd parity: the third parity; Average: the average of litter size. The H-value is expressed as a test statistic and is used to measure differences between groups.

**Table 5:** Association of *GDF9* gene polymorphisms and litter size in Hu sheep.

Locus	Parity	Genotype	Number of Samples	Mean (Median)	H-Value	*p*-Value
*GDF9*-G2 C > T [18]	1st	CT	4	2.600 (2.000)	0.052	0.891
		CC	187	2.436 (2.000)		
	2nd	CT	4	2.000 (1.500)	0.358	0.358
		CC	165	2.321 (2.000)		
	3rd	CT	4	2.500 (2.500)	0.801	0.801
		CC	135	2.370 (2.000)		
	Average	CT	4	2.415 (2.165)	0.088	0.767
		CC	187	2.367 (2.330)		
*GDF9*-G3 G > A [18]	1st	AA	16	2.313 (2.000)	0.543	0.762
		GA	97	2.505 (2.000)		
		GG	78	2.313 (2.000)		
	2nd	AA	15	2.067 (2.000)	0.942	0.624
		GA	84	2.345 (2.000)		
		GG	70	2.329 (2.000)		
	3rd	AA	12	2.417 (2.000)	0.066	0.967
		GA	69	2.348 (2.000)		
		GG	58	2.397 (2.000)		
	Average	AA	16	2.289 (2.170)	1.072	0.585
		GA	97	2.382 (2.330)		
		GG	78	2.345 (2.330)		
*GDF9*-G4 G > A [18]	1st	GA	30	2.400 (2.000)	0.076	0.783
		GG	161	2.447 (2.000)		
	2nd	GA	29	2.276 (2.000)	0.085	0.770
		GG	140	2.321 (2.000)		
	3rd	GA	26	2.115 (2.000)	0.933	0.164
		GG	113	2.434 (2.000)		
	Average	GA	30	2.277 (2.330)	0.137	0.711
		GG	161	2.385 (2.330)		

Note: 1st parity: the first parity; 2nd parity: the second parity; 3rd parity: the third parity; Average: the average of litter size. The H-value is expressed as a test statistic and is used to measure differences between groups.

## Data Availability

The data presented in this study are available on request from the corresponding author.

## Supplementary Materials

The following are available online at https://www.mdpi.com/article/10.3390/genes16020168/s1, Figure S1: Histogram of normal distribution of BMP15 c.31_33CTTdel locus, Figure S2: Histogram of normal distribution of GDF9 (G2, G3, and G4) locus.
