# Supplementary material for: Association of BMP15 and GDF9 Gene Polymorphisms with Litter Size in Hu Sheep
Source: Genes (Basel). 2025 Jan 28;16(2):168. doi: 10.3390/genes16020168 (PMC11855564; doi:10.3390/genes16020168)
Supplement: Supplementary file 1 [file genes-16-00168-s001.zip › genes-3430081-supplementary.pdf]

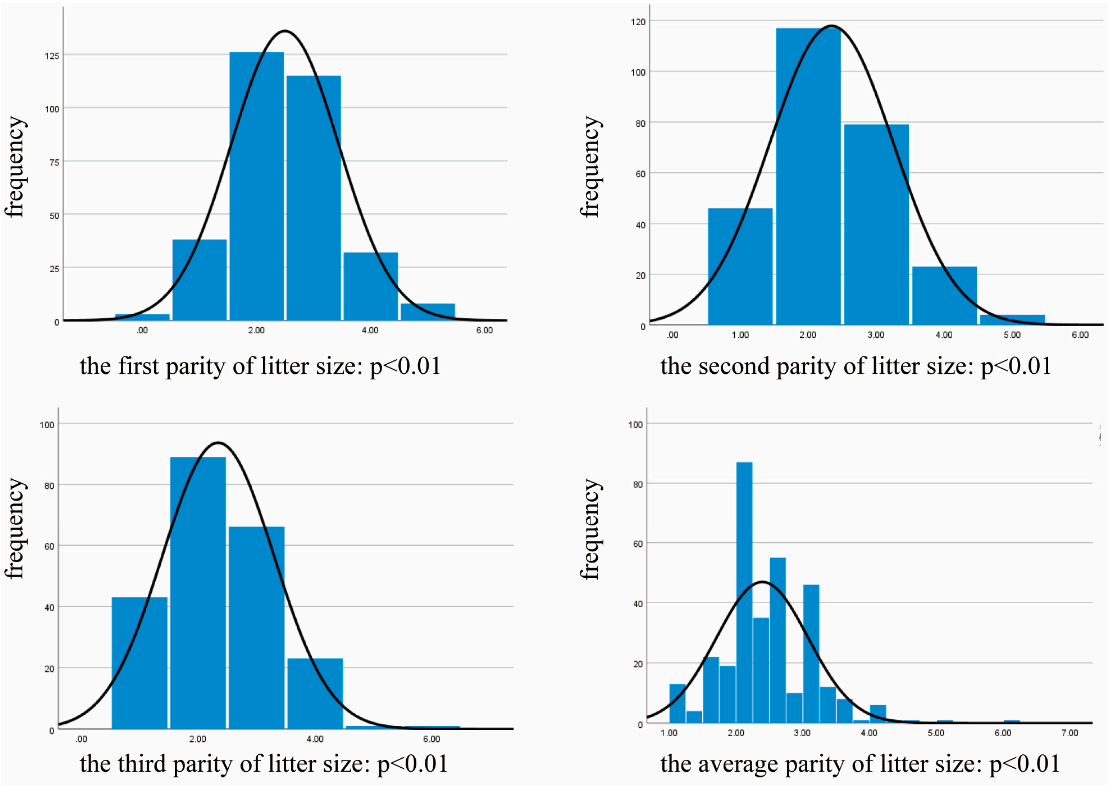

Figure S1: Histogram of normal distribution of *BMP15* c.31_33CTTdel locus. Note: p<0.05, does not conform to normal distribution.

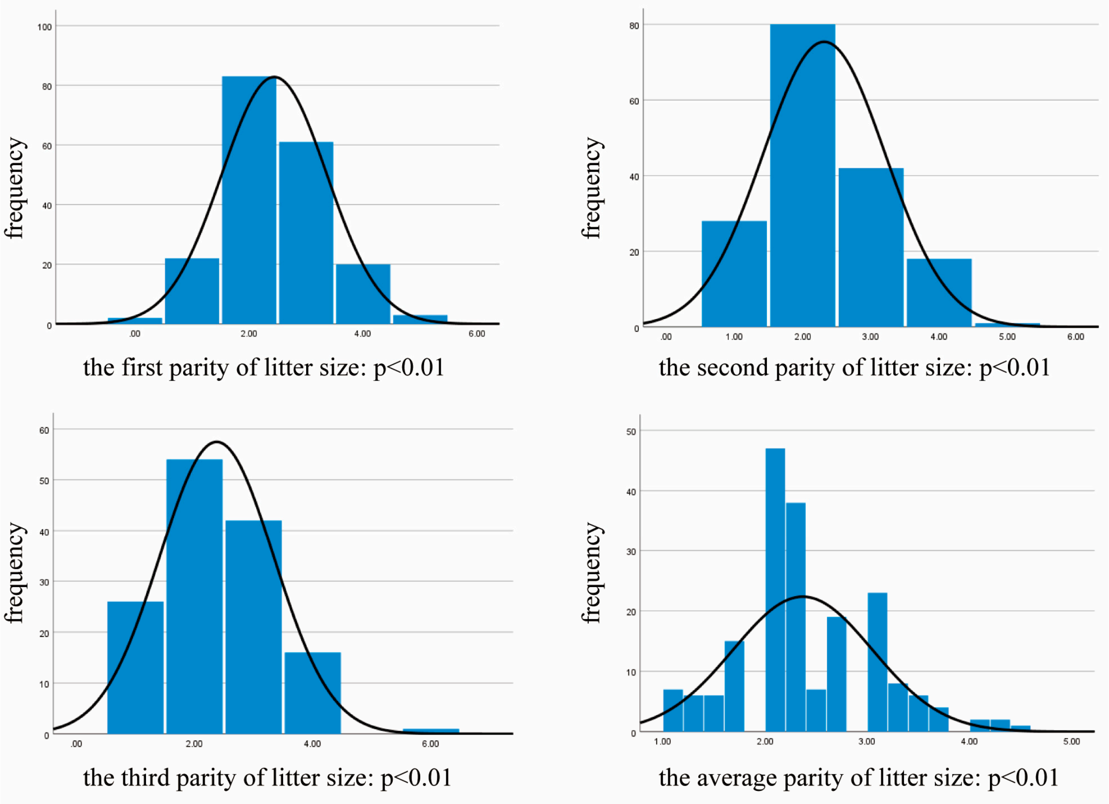

Figure S2: Histogram of normal distribution of *GDF9* (G2,G3,G4) locus. Note: p<0.05, does not conform to normal distribution.
